# Dynamic stretching alone can impair slower velocity isokinetic performance of young male handball players for at least 24 hours

**DOI:** 10.1371/journal.pone.0210318

**Published:** 2019-01-25

**Authors:** Monoem Haddad, Mohammad Shoaib Prince, Nidhal Zarrouk, Montassar Tabben, David G. Behm, Karim Chamari

**Affiliations:** 1 Sport Science Program, College of Arts and Sciences, Qatar University, Doha, Qatar; 2 National Sports Medicine Programme, Excellence in Football Project, Aspetar - Orthopedic and Sports Medicine Hospital, Doha, Qatar; 3 ASPREV Department, Aspetar Orthopedic and Sports Medicine Hospital, Doha, Qatar; 4 School of Human Kinetics and Recreation, Memorial University of Newfoundland, St. John’s, Newfoundland, Canada; 5 AHP Research Centre, Aspetar, Qatar Orthopaedic and Sports Medicine Hospital, Doha, Qatar; Catholic University of Brasilia, BRAZIL

## Abstract

There are many adult studies reporting static stretch (SS)-induced deficits and dynamic stretch (DS) performance improvements shortly after the intervention. However, there is only a single study examining stretch-induced performance changes with youth at 24 hours’ post-stretch. The objective of this study was to examine physiological responses of young trained athletes at 24-hours after experiencing SS or DS protocols. Eight young male, elite handball players (age: 16.1±5.1 years) were tested prior to-, 3-minutes and 24-hours following the three conditions (DS, SS, Control) in a randomized and counterbalanced order. Similar volumes of SS (2 repetitions of 75s for each leg) and DS (5 repetitions of 30s for each leg) involved one stretch each for the quadriceps and hamstrings. Tests included (i) two 4s maximal voluntary isometric contractions (MVC) at 60° of knee flexion with 2-min rest, (ii) two maximal isokinetic contractions each at 60°/sec and 300°/sec with 1-min rest, and (iii) two drop jumps with 30-sec rest. To simulate a full warm-up, dynamic activity including 5 minutes of aerobic cycling (70 rpm; 1 kilopond), 4 submaximal isometric contractions and 4 drop jumps were instituted before the pre-tests and following the interventions. Two-way repeated measures ANOVAs revealed that 1) both the SS and control conditions exhibited knee extensor 60°.s^-1^ (SS:-10.3%; p = 0.04, Control: -8.7%; p = 0.07) and 300°.s^-1^ (SS: -12.9%; p = 0.005, Control: -16.3%; p = 0.02) isokinetic deficits at post-test, 2) DS impaired knee flexor 60°.s^-1^ isokinetic work and power-related measures at post-test (Work: -10.1%; p = 0.0006; Power: -19.1%; p = 0.08) and at 24-hours’ post-test (Work: 9.9%; p = 0.023; Power: -9.6%; p = 0.01), 3) DS (12.07% and 10.47%) and SS (13.7% and 14.6%) enhanced knee flexor 300°.s^-1^ isokinetic force and power-related measures compared to control. In conclusion, testing-induced knee extensor isokinetic impairments were counterbalanced by DS, however the hip flexion DS could have produced minor muscle damage for at least 24-hours decreasing knee flexor forces and power at 60°.s^-1^.

## Introduction

Prolonged static stretching (SS) has been reported to impair subsequent performance [1–3]. The time course of impairments with SS can range from 2- [4, 5], 5- [6–8], 10- [9–14], 20- [15], 60- [16] and 120-minutes [17]. However, in some studies, deficits reported soon following SS are not apparent shortly thereafter. For example, Bradley et al. [8] reported countermovement jump (CMJ) deficits at 5 minutes, which were not present at 15 minutes post-stretching. Two main mechanisms purported to be responsible for this performance reduction with acute static stretching involve (i) neurological inhibition, leading to a decrease in neuromuscular activation or the reflex sensitivity [1, 2, 18] and (ii) mechanical factors, causing a decrease in the stiffness of the musculotendinous unit (MTU) that can affect the length-tension relationship of the muscle [4, 19, 20].

In contrast, dynamic stretching (DS) is often reported to enhance or have no significant impairment effects on subsequent performance [1, 2]. DS-induced increases in performance (i.e. jump height, 1-RM, isokinetic torque, sprint, agility) have been shown to continue for 2- [21–25], 5- [26–29], 10- [10, 30, 31] and 20-minutes post-stretching [32, 33]. Although there are more studies reporting either no change or improvements in performance post-DS, not all DS demonstrate performance improvements. Indeed, DS-induced impairments have been reported at 5- [34], 10- [35], and 30-minutes post-intervention [26]. One major limitation of all the stretching-related studies is that the measures were typically conducted immediately after testing or between 2–30 minutes after stretching. Very little is known about the longer duration acute effects of SS and DS.

Prolonged mechanical stretching of the musculotendinous unit may induce damage reducing muscle force production [36], but the extent of this damage may not be practically meaningful in adults [1]. Acute muscle stretching can reduce tendon stiffness, forcing the muscle to work at shorter, and weaker (force-length relationship), lengths [16, 37–39]. Impairments in the efficiency of the electromechanical transfer of force may be induced by stretch-induced changes in endo-, epi- and perimysial transmission [40] as well as the stretch-induced reductions in muscle stiffness [3, 41]. Mechanical changes and disruptions may not resolve rapidly and could influence performance for days. However, while this research has been conducted in animals and adult humans, the consequences of prolonged effects from stretch-induced changes in muscle and connective tissue have not been examined with youth.

Haddad et al. [42] is the only study to demonstrate SS-induced performance impairments and DS-induced performance enhancements at 24 hours post-stretching. Their protocol employed 17–19 year old elite soccer players who stretched either with SS or DS for 2 sets of 7 minutes: 30 seconds each for 5 muscle groups (each muscle group received 30s for the right and 30s for the left side with 15s recovery between repetitions and a 3-minute recovery between sets). Since their measures involved athletic performance such as repeated sprints, 30-meter sprint and jump performance, there is also a need to examine more specific physiological measures.

The preponderance of stretching studies have used adult participants and studies are needed examining the response of youth to stretching protocols. Indeed, the response of youth to stretching can differ. For example, Chaouachi et al. [43] reported that stretch training reduced acute SS-induced impairments, whereas stretch training with adults did not attenuate acute SS deficits [6]. With only one published study [42] investigating prolonged acute effects (24 hours post-stretching), further research is also needed to substantiate these earlier findings [44]. The objective of the present study was therefore to examine responses of adolescent male trained athletes at 24 hours after experiencing SS or DS protocols. Based on the prior work by Haddad et al. [42], it was hypothesized that SS and DS-induced impairments and enhancements would occur, respectively.

## Methods

### Experimental design

The young athletes were involved with three experimental conditions: SS, DS, and control (without stretching), in a randomized, counter-balanced order. The effect of the conditions on subsequent physiological measures was monitored with isometric and isokinetic maximal voluntary contraction (MVC) forces and electromyography (EMG) measurements of the quadriceps and hamstrings at 24-h post-stretching.

### Subjects

Eight young handball players (age: 17.33±1.07 years (under 19 years team), mass: 78.6±14.5 kg, height: 1.83±0.07 m, BMI: 23.4±3.7, % body fat: 3.8±0.5, experience: 7.83±1.34 years) from the same team volunteered to participate in the study. All eight players performed the three conditions and thus were included in the statistical analysis. No musculoskeletal injuries were reported in the 4 weeks prior to the beginning of the study. Players participated in handball training on average 10.1±1.1 hours/week and 1–2 matches a week but they did not train between the pre-test and post-24 hour tests. Prior to the study, all players and parents were informed about the potential risks and benefits associated with participation. All players and their parents/guardians signed informed consent forms. The athletic participants were fully familiarized with the procedures and they were informed that they could withdraw from the study at any time without penalty. This study was approved by the national university institutional review board for human subjects and complied with requirements for Declaration of Helsinki.

### Procedure

Data were collected over 4-weeks (March-April 2017) during the competitive season (August-May). In the first week, all players attended 2 orientation sessions on the same day. The first session was dedicated to anthropometric measurements. The second session involved familiarization with all tests and procedures. Detailed information about stretching and strength measurements were provided. All players performed a familiarization trial to practice the three tests: 1) isometric MVC at 60° of knee flexion, 2) isokinetic knee extensor and flexor strength measurements in concentric modes at two selected angular velocities (60°∙s^-1^ and 300°∙s^-1^) and 3) drop jump. EMG activities were recorded during the familiarization session to ensure the youth were familiarized and comfortable with the material and conditions.

In the following 3-weeks, players performed one of the three conditions in a counterbalanced order. A Latin Squares, three-condition research program was used. This design is a compromise, designed to balance the strengths of counterbalancing with financial and practical reality. It attempts to circumvent some of the complexities and keep the experiment to a reasonable size.

Each week included a pre-test session (day 1—DS, SS, Control), post-test, and a 24-h post-test session (day 2). Tests included strength measures: (i) two isometric MVCs (4 second duration) at 60° of knee flexion (a third contraction was performed if more than 5% difference in peak force was observed in the two first trials) with 2-min rest, (ii) two maximal isokinetic contractions at 60°.s^-1^ with 1-min rest, and (iii) two isokinetic contractions at 300°.s^-1^with 1-min rest and, two drop jumps with 30-seconds’ rest. Two-minutes’ rest was provided between the pre-test and the intervention (see further).

A reported limitation of many stretching studies is that the stretching is performed in isolation and a full warm-up has not been incorporated [1]. Thus in order to simulate a full warm-up, a dynamic aerobic activity was instituted before the pre-tests and following the interventions. Prior to the pre-test, and after the intervention, each player performed a warm-up on a stationary cycle ergometer for 5-minutes at 70 rpm and 1 kilopond. The warm-up also contained four submaximal contractions at approximately 50–75% of MVC for 4-seconds each and 10-seconds’ rest between repetitions, followed by 3 drop jumps at 75% of their best jump height with 15-30-seconds’ rest between repetitions. There was no SS in the warm up performed immediately before the tests. The same prior warm up and testing were used after 24-hours. Post-tests were conducted 3-minutes after the cycling to prevent any potential impact of fatigue. No training was permitted between post-test and 24-h post-test with the participants’ coach being part of the process. The latter was present at all testing sessions to ensure the participants exercised at their maximal capacity and also to ensure they did not perform any kind of training sessions that would have impacted the results of the study.

For diet monitoring, each player was given a meal plan (food and hydration) composed in collaboration with the club’s nutritionist. The day before testing, they were prohibited from consuming any known stimulant (e.g., caffeine) or depressants (e.g., alcohol) substance. To avoid dehydration, ad libitum drinking was permitted during all training sessions.

#### Stretching protocols (intervention)

The SS and DS stretching protocols were used in previous studies (Turki et al. 2011; Haddad et al. 2013). The same duration was instituted for each muscle group in the two stretching protocols.

**Static stretching (SS)**: The SS involved lengthening the muscle until the point of discomfort was reached [7, 45, 46] and then the muscle was maintained in the lengthened position for two sets of 75-sec each for each muscle group [9, 47]. SS exercises included the following:

Quadriceps: Standing knee flexion and hip extension stretch: The participant stood on one foot facing a wall, with the foot-in-hand on the same side, one hand resting on the wall for balance. The heel was placed in contact with the buttocks until discomfort was felt, and remained in that position while keeping the back straight without moving the thigh. If touching the buttocks with flexed knee alone did not provide sufficient stress on the quadriceps, the participant was urged to extend the hips (knee moves posterior or dorsal to the frontal plane of the body).
Hamstrings: Sit and reach stretch for hamstrings and lower back. The participant sat on the floor, with legs straight, and ankles dorsiflexed, attempting to contact and hold the feet with the hands, leaving the lower limbs straight (if possible).

**Dynamic stretching (DS)**: All players performed five repetitions of 30-seconds of DS for the quadriceps and hamstrings for each leg (30-seconds right leg / 30-seconds left leg) through a full, but not painful range of motion at a high speed while still maintaining control [1, 2, 48]. Hence the motion was not ballistic.

Quadriceps: Butt kicks: The participant performed quick kicks to the buttocks (knee flexion) as they moved forward with one kick per second alternating the legs.

Hamstrings: Dynamic hip flexion stretch: The participant kicked the legs forwards (hip flexion) at an angle greater than 90°, while keeping the knees in full extension at a rate of one kick per second. The action was performed in a controlled manner to decrease the chances of injuries from uncontrolled ballistic movements.

#### Isokinetic and isometric testing

Knee extension strength (force) was measured with an isokinetic dynamometer (Isoforce, TUR Gmbh, Germany). Participants sat on the dynamometer with their hips, thighs, and upper body firmly strapped to the seat. In this position, their hip angle was at 100° of flexion. The lower leg (left leg) was then attached to the arm of the dynamometer at a level slightly above the lateral malleolus of the ankle joint, and the axis of rotation of the dynamometer arm was aligned with the lateral femoral condyle. The dynamometer arm was then set at 60° from full knee extension. Each player performed four submaximal familiarization contractions before performing two MVCs; the highest force contraction at 60° was used for normalization of EMG data. All participants were verbally and vigorously encouraged to exert maximal effort during both MVCs. The peak force and the force exerted within the first 100ms of effort (instantaneous strength) were analyzed [10, 49, 50]. The prolonged effects of stretching on velocity specific isokinetic torque were also examined. Players performed two contractions at 60°.s^-1^ and two contractions at 300°.s^-1^.

#### EMG measurements

Since stretch-induced performance impairments have been partially attributed to neuromuscular activation impairments (Behm and Chaouachi 2011, Kay and Blazevich 2012, Behm et al. 2016), muscle activation measured with EMG activity (Behm et al. 2001b, 2004, 2011b, Costa et al. 2014, Fowles et al. 2000, Hough et al. 2009, Janes et al. 2016, Power et al. 2004) was monitored from the isometric MVC trial with the highest force output and for all the isokinetic contractions. MVC EMG data were recorded before the first set of isokinetic contractions for both conditions to ensure similar normalization of EMG in the two trials. During the MVC measurements, EMG activity was recorded and sampled at 2000 Hz with sensors placed on the three heads of the quadriceps muscle (rectus femoris, vastus medialis, vastus lateralis) and hamstrings (semimembranosus, semitendinosus) [20] using Delsys Trigno Wireless EMG System (Delsys, Boston, MA, USA). EMG skin preparation and electrode placement followed SENIAM recommendations [51]. Consequently, the Delsys EMG system streamed the data digitally into EMGworks software. The software stored and expressed the raw EMG data as absolute root-mean-square amplitude values (mV).

**Drop Jump**: After EMG and MVC measurements, a drop jump test using a mat equipped with photoelectric cells (Optojump, Microgate, BZ, Italy) was performed pre-, post- and 24-h after performing the different stretching conditions to assess jump height, contact time and reaction force. Participants were instructed to jump as high as possible with the shortest possible time on the floor.

### Statistical analyses

Statistical analyses were computed using the SPSS software (Version 23.0, SPSS, Inc, Chicago, IL, USA). The assumption of sphericity and normality was tested for all dependent variables. The assumptions were satisfied for each data set. Reliability was assessed with Cronbach's alpha intraclass correlation coefficient (ICC) measurements from the pre- and post-test values from the control session. Measures were analyzed with a 2-way repeated measures ANOVA (3 conditions x 3 times) to identify specific main effects and interactions. When the F value was significant, a Bonferroni post-hoc comparisons were conducted for main and interaction effects. Cohen’s *d* effects sizes (ES)[52] were also calculated by SPSS software to determine the magnitude of the differences between main effects. To identify specific significant interactions the Cohen’s effect size calculation was employed as follows: Effect Size = (M2—M1) / SDpooled. The following criteria were used: ES < 0.2 was classified as trivial, 0.2–<0.5 was considered small; 0.5–<0.8 represented medium; and ≥0.8 represented a large effect size. Minimally clinically important or meaningful differences can be observed by examining the standard error of the mean (SEM) or whether the difference is classified as a trivial effect size (<0.2). Since the SEM is the variation in scores due to unreliability of the measure, a change that is less than the SEM is likely due to measurement error rather than a true observed change (11).

*Reliability*: Table 1 illustrates the generally excellent reliability of most measures in the present study. Time to peak torque and MVC rectus femoris EMG activity were the only measures that consistently ranged between ~0.7–0.8 (acceptable range).

**Table 1:** Reliability with Cronbach alpha intraclass correlation coefficients (ICC) and standard error of the means (SEM), RMS: Root mean square.

Measure	Cronbach Alpha ICC	Standard Error of the Measurement: Control, DS, SS Conditions
Knee Extensor Isokinetic 300°.^s-1^		
Peak Torque	0.963	8.8–9.75
Peak Torque / kg (relative)	0.981	0.25–0.27
Time to Peak Torque	0.730	0.047–0.068
Mean Power	0.947	29.3–29.8
Power / kg (relative)	0.977	0.66–0.76
Maximum Work	0.947	12.9–14.2
Work / kg (relative)	0.968	0.328–0.338
Total Work	0.947	12.9–14.3
Knee Flexor Isokinetic 300°.^s-1^		
Peak Torque	0.876	5.4–7.5
Peak Torque / kg (relative)	0.935	0.19–0.24
Time to Peak Torque	0.690	0.23–0.24
Mean Power	0.836	10.1–17.5
Power / kg (relative)	0.864	0.19–0.42
Maximum Work	0.916	8.7–12.8
Work / kg (relative)	0.927	0.19–0.28
Total Work	0.916	9.1–12.8
Knee Extensor Isokinetic 60°.^s-1^		
Peak Torque	0.951	13.6–18.8
Peak Torque / kg (relative)	0.694	4.1–5.1
Time to Peak Torque	0.733	0.022–0.036
Mean Power	0.964	9.9–11.5
Power / kg (relative)	0.688	0.16–0.48
Maximum Work	0.946	16.5–20.4
Work / kg (relative)	0.962	0.40–0.55
Total Work	0.902	16.5–20.3
Knee Flexor Isokinetic 60°.^s-1^		
Peak Torque	0.857	5.5–12.6
Peak Torque / kg (relative)	0.958	0.17–0.27
Time to Peak Torque	0.657	0.044–0.048
Mean Power	0.620	5.8–10.6
Power / kg (relative)	0.720	0.08–0.27
Maximum Work	0.921	11.4–15.7
Work / kg (relative)	0.909	0.27–0.29
Total Work	0.766	11.4–26.3
MVC Electromyography (EMG)		
Vastus Lateralis RMS EMG	0.817	0.016–0.027
Rectus Femoris RMS EMG	0.729	0.017–0.026
Vastus Medialis RMS EMG	0.855	0.021–0.032

## Results

### Knee extensors

**Fig 1:**
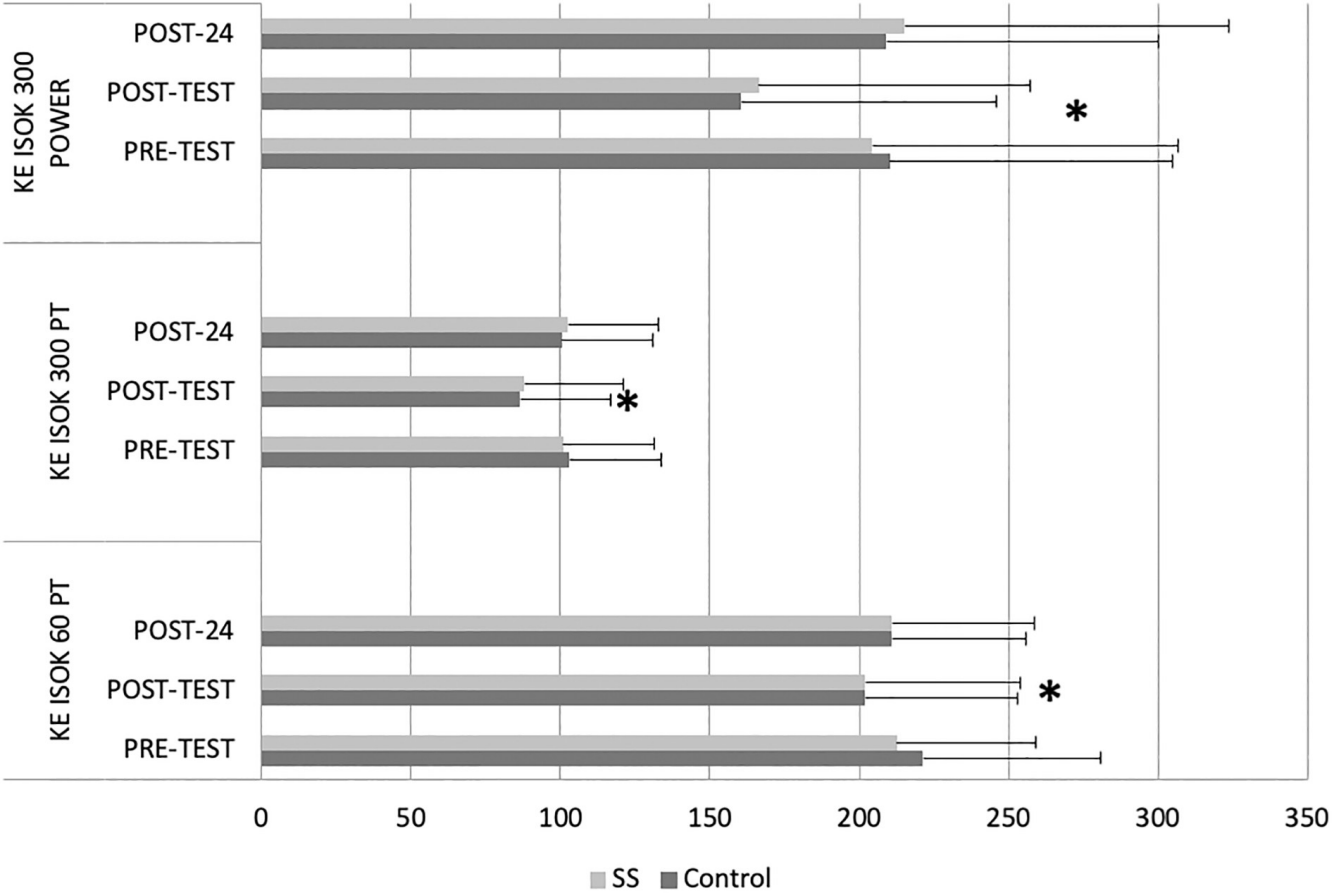
Figure illustrates mean ± standard deviation knee extensors (KE) condition (static stretching (SS) and control) x time (pre-test, post-test and post-24 hours test) interactions with significant differences between all pre- and post-tests (*). KE: Knee extensors, ISOK: isokinetic, PT: Peak torque (kg.metres), 60: isokinetic contraction velocity of 60°.s^-1^, 300: isokinetic contraction velocity of 300°.s^-1^, Power (kg.s^-1^).

Main effects for time exhibited prolonged knee extension MVC time to peak torque, decreases in knee extension isokinetic peak torque at 60°.s^-1^, and 300°.s^-1^ as well as relative peak torque, mean absolute and relative power and work, and total work at 300°.s^-1^ (Table 1). Significant interactions illustrated that both control and SS conditions experienced pre- to post-test decrements in knee extension isokinetic peak torque at 60°.s^-1^, and 300°.s^-1^ as well as relative peak torque, mean absolute and relative power at 300°.s^-1^ (Table 2). Fig 1 illustrates mean ± standard deviation knee extensors condition (static stretching (SS) and control) x time (pre-test, post-test and post-24 hours test) interactions with significant differences between all pre- and post-tests.

**Table 2:** Knee extension (KE) time main effects and condition x time interactions impairments all occurred at post-test.

Measures	Time Main effects	Interaction Control @ post-test	Interaction Static Stretch @ post-test
Knee Extensor MVC Time To Peak Torque	F_(2, 4.08);_ p = 0.041; -17.8% d = 0.31 Power = 0.65		
Knee Extensor Isokinetic 60 Peak Torque (Pt)	F_(2, 8.37);_ P = 0.003 -6.8% d = 0.48 Power = 0.93	p = 0.07 -8.7% ES = 0.45 Power = 0.61	p = 0.04 -10.3% ES = 0.28 Power = 0.61
Knee Extensor Isokinetic 300 Peak Torque	F_(2, 10.43);_ p = 0.001 -10.8% d = 0.54 Power = 0.97	p = 0.02 -16.3% ES = 0.54 Power = 0.60	p = 0.005 -12.9% ES = 0.42 Power = 0.60
Relative Knee Extensor Isokinetic 300 Pt (Pt/Kg)	F_(2, 11.07);_ p = 0.001 -10.1% d = 0.55 Power = 0.98	p = 0.009 -14.9% ES = 0.38 Power = 0.60	p = 0.02 -11.7% ES = 0.28 Power = 0.60
Knee Extensor Isokinetic 300 Power	F_(2, 12.13);_ p = 0.0001 -16.5% d = 0.57 Power = 0.98	p = 0.02 -23.7% ES = 0.53 Power = 0.57	p = 0.004 -18.5% ES = 0.37 Power = 0.57
Relative Knee Extensor Isokinetic 300 Power (Power/Kg)	F_(2, 13.63);_ p = 0.0001 -15.8% d-0.60 Power = 0.99	p = 0.008 -22.6% ES = 0.40 Power = 0.60	p = 0.006 -17.7% ES = 0.28 Power = 0.60
Knee Extensor Isokinetic 300 Mean Work	F_(2, 7.93);_ p = 0.03 -12.6% d = 0.47 Power = 0.92		
Relative Knee Extensor Isokinetic 300 Work (Work/Kg)	F_(2, 8.89);_ p = 0.002 -11.8% d = 0.49 Power = 0.94		
Knee Extensor Isokinetic 300 Total Work	F_(2, 7.91);_ p = 0.003 -12.6% d = 0.47 Power = 0.92		

LE: Leg extension, ISOK: isokinetic, PT: peak torque, 60: 60°.s^-1^, 300: 300°.s^-1^, d = ETA^2^.

There were no significant main effects or interactions with vastus lateralis, rectus femoris or vastus medialis RMS EMG activity during the MVC tests.

### Knee flexors

Significant interactions showed that knee flexors isokinetic absolute and relative peak torque, work and absolute mean power at 60°.s^-1^ were impaired pre- to post-test following dynamic stretching (Table 3). In addition, significant pre- to post-24 hour deficits were apparent with isokinetic absolute and relative work and absolute mean power at 60°.s^-1^ (Table 3). Furthermore, a main effect for condition revealed significant and near significant lower absolute (p = 0.05) and relative (p = 0.08) knee flexor isokinetic 300°.s^-1^ mean power for control versus dynamic and static stretching conditions respectively (Table 4).

**Table 3:** Knee flexion (KF) condition x time interaction impairments with dynamic stretching.

Measure	Interaction	Pre- vs. Post	Pre- vs. Post-24
Knee Flexor Isokinetic 60 Peak Torque (Pt)	F_(2, 9.71);_ p = 0.002 d = 0.37 Power = 0.95	p = 0.00021 -8.5% ES = 0.52	
Relative Knee Flexor Isokinetic 60 Pt (Pt/Kg)	F_(2, 7.45);_ p = 0.035 d = 0.24 Power = 0.76	p = 0.00027 -8.1% ES = 0.29	
Knee Flexor Isokinetic 60 Mean Power	F_(2, 6.85);_ p = 0.0 d = 0.22 Power = 0.66	p = 0.08 -19.1% ES = 0.94	p = 0.01 -9.6% ES = 0.47
Knee Flexor Isokinetic 60 Work	F_(2, 7.63);_ p = 0.05 d = 0.22 Power = 0.65	p = 0.0006 -10.1% ES = 0.46	p = 0.023 -9.9% ES = 0.45
Relative Knee Flexor Isokinetic 60 Work (Pt/ Kg)	F_(2, 8.89);_ p = 0.029 d = 0.25 Power = 0.75	p = 0.001 -9.7% ES = 0.37	p = 0.05 -8.6% ES = 0.34

ISOK: isokinetic, PT: peak torque, d = ETA^2^, ES = effect size

**Table 4:** Table illustrates a main effect for condition with the control condition being significantly (*) lower than the dynamic and static stretch conditions when testing with isokinetic 300°.s^-1^.

MAIN EFFECT FOR CONDITION	Control	Dynamic Stretch	Static Stretch
Knee Flexor Absolute Power (Kg.s^-1^)	148.5±41.6 * F_(2, 8.01);_ p = 0.05	168.8±42.1 13.6% ↑ d = 0.48	172.3±40.3 16.1%↑ d = 0.58
Knee Flexor Relative Power (Kg.s^-1^/kg)	2.99±1.3 * F_(2, 5.94);_ p = 0.08	3.34±0.95 11.7%↑ d = 0.35	3.5±1.1 17.1%↑ d = 0.42

**Fig 2:**
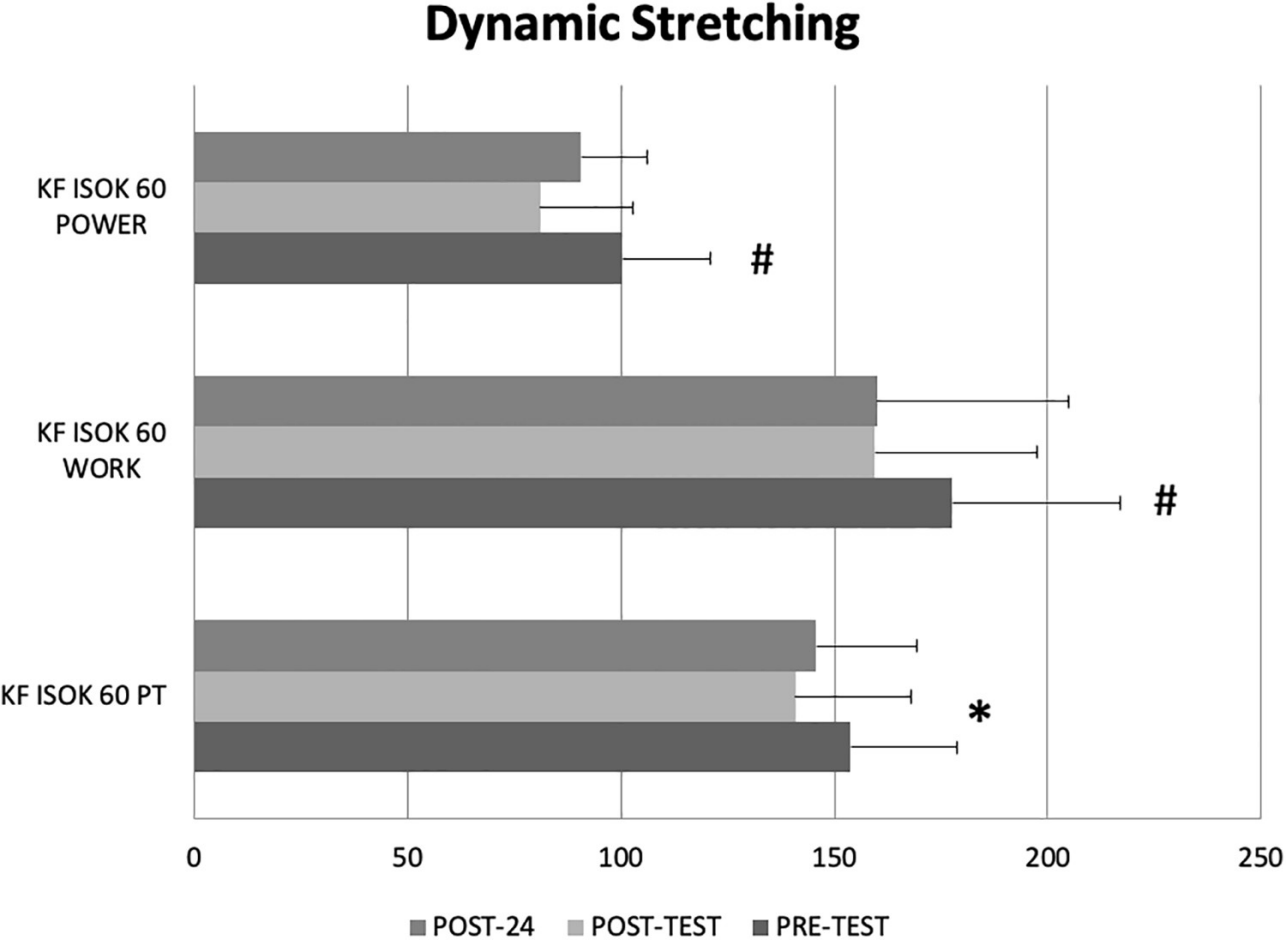
Figure shows mean ± standard deviation knee flexor (KF) dynamic stretching resulted in significant decreases from pre- to post-test for isokinetic (ISOK) peak torque (PT: kg.metres) at a contraction velocity of 60°.s^-1^ (*) as well as significant decreases for both post-test and 24 hours’ post-test (POST-24) compared to pre-test (#) for total work (Joules) and mean power (kg.s^-1^).

Fig 2 shows mean ± standard deviation knee flexor dynamic stretching resulted in significant decreases from pre- to post-test for isokinetic peak torque at a contraction velocity of 60°.s^-1^ as well as significant decreases for both post-test and 24 hours’ post-test (POST-24) compared to pre-test for total work and mean power.

There were no significant main effects or interactions with semimembranosus or semitendonosus RMS EMG activity during the MVC tests.

### Drop jump height

**Fig 3:**
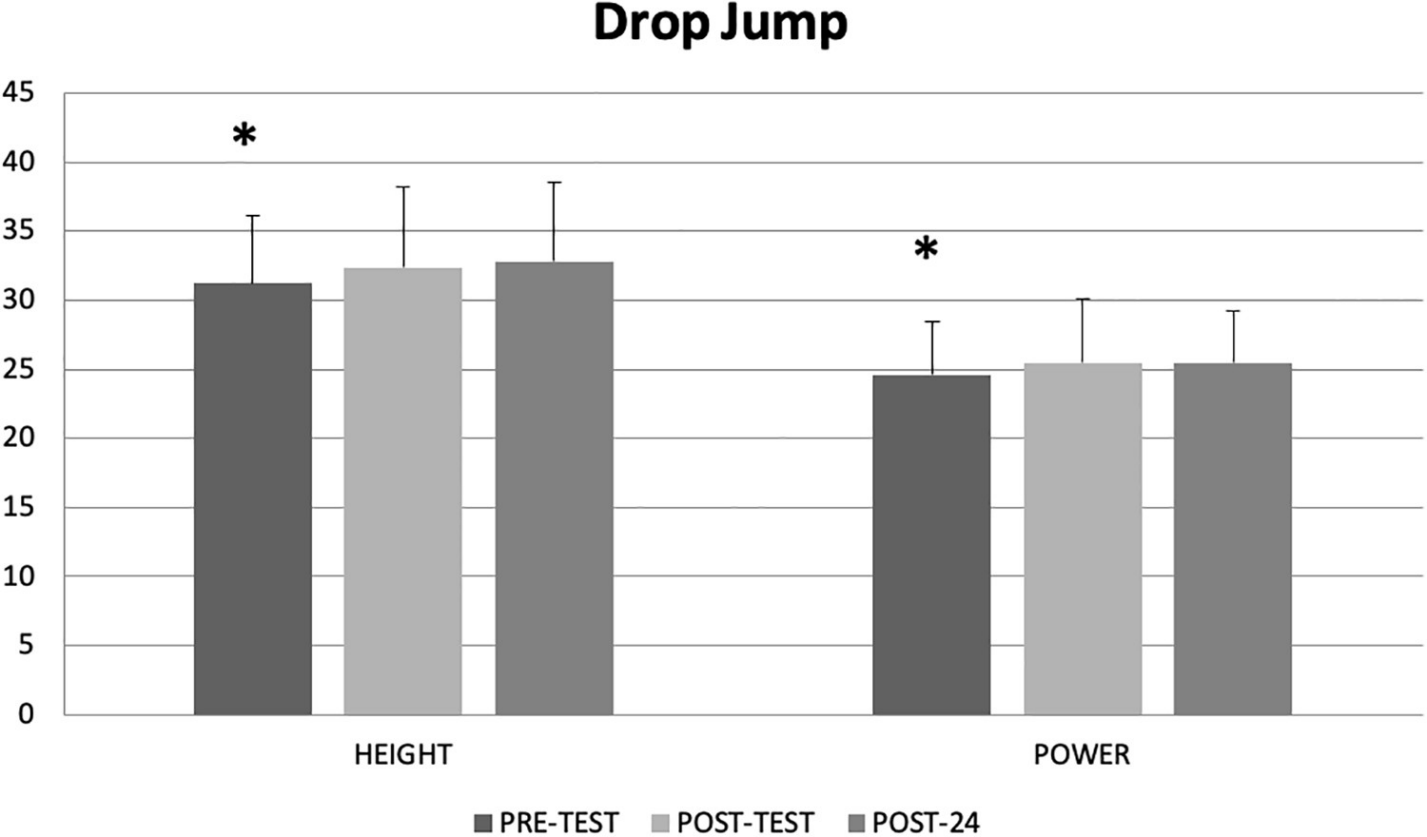
Figure illustrates a main effect for time with the pre-test demonstrating lower drop jump height (cm) and power (kg.s^-1^) than both the post-test and 24 hours’ post-test (POST-24). Means ± standard deviations are illustrated.

Main effects for time demonstrated significant increases in drop jump power (p = 0.05, p = 0.28) and height (p = 0.008, d = 0.41) at post-test (3.5% and 3.6%) and post-24 hours (3.5% and 4.9%) (Fig 3).

## Discussion

The most important findings of the present study were that 1) both the SS and control conditions exhibited 60°.s^-1^ and 300°.s^-1^ knee extensors isokinetic force and power-related deficits at post-test, 2) DS impaired 60°.s^-1^ knee flexors isokinetic force and power-related measures at post-test and 24 hours’ post-test, 3) DS and SS enhanced 300°.s^-1^ knee flexors isokinetic force and power-related measures compared to control (main effect for condition).

The SS and control condition decrements in 60°.s^-1^ and 300°.s^-1^ knee extensors isokinetic force and power-related deficits at immediately post-test would indicate that the testing (i.e. since control condition decreased as well) may have contributed to this performance depression. With the demands of competitive handball, the youth participants would be expected to possess a relatively high degree of lower body muscular power (from sprints, agility/change of direction and kicking), aerobic capacity, and muscular endurance (continuous movement over a 60-minute match). However, exerting maximal forces on a dynamometer with isokinetic and isometric contractions even with familiarization sessions may have presented novel challenges to their neuromuscular system. The intent to perform maximal contractions may be compromised in youth to a lack of experience to this contraction intensity, testing devices or the neurological immaturity of the youth [53–55]. However, this compromise is unlikely since with a familiarization session and a counterbalanced randomized allocation of testing, the adolescents would have had 10 opportunities throughout the experiment to perform maximal isokinetic and isometric contractions. Furthermore, the reliability of the MVC tests were typically high. Although two minutes of rest was provided between the post-stretch intervention second cycling bout and post-testing, it might be possible that the combination of isokinetic, MVC testing, countermovement jump pre-tests and cycling could have contributed to small magnitude post-test deficits that were offset by dynamic stretching.

In contrast to the SS and control-induced post-test deficits, the lack of significant DS knee extension impairments at post-test, suggests the dynamic movements counterbalanced the control and SS-induced post-test force deficits. A number of adult DS studies report subsequent improved force and power development and athletic performance (i.e. jump height) [25, 29, 56–58]. The Behm et al. [1] meta-analysis indicated that the weighted mean performance enhancement associated with 48 adult DS studies was 1.3% ± 4.7%. The mechanisms related to these performance enhancements could be attributed to DS-related elevation of core temperature [59], increased nerve conduction velocity, muscle compliance, enzymatic cycling, [60], induction of post-activation potentiation in the stretched muscle [25, 32, 57], increased central drive [1, 2] and decreased inhibition of antagonist muscles [29, 61].

However, DS impaired 60°.s^-1^ knee flexors isokinetic force and power-related measures at post-test and 24 hours’ post-test. Whereas Haddad et al. [42] was the first study to report SS-induced decrements 24 hours after a bout of SS, this is the first study to show DS impairments at 24 hours post-DS. One of the DS exercises was a swinging hip flexion and extension motion. The range of motion is considerably greater with hip flexion than with hip extension [62]. Even with proper execution, if this hip flexion ROM exceeded the participants typical ROM then, it might be possible that a minor, undetected, musculotendinous strain could be induced. Although the participants were monitored by researchers and the coach during the DS protocol, it is possible that some of the participants in the enthusiasm and competitiveness of a team group setting could have kicked their legs anteriorly to such a degree that some minor hamstrings muscle damage occurred inhibiting subsequent isokinetic force and power at an angular speed of 60°.s^-1^. Further evidence for possible peripheral or muscle damage is the lack of change in EMG activity at post-test or 24 hours’ post-test. A muscle strain or delayed onset muscle soreness would not be expected to fully recover in 24 hours [63, 64]. Whereas Power et al. [17] reported non-significant evoked contractile property force decrements two hours after stretching, Behm et al. [9] and Fowles et al. [16] demonstrated approximately 12% and 18% decreases in evoked twitch force respectively. Impairments in evoked force can reflect impairments to myofilaments or excitation contraction coupling, which might take days to resolve. Furthermore, DS-induced impairments have also been attributed to decreased musculotendinous unit passive stiffness and passive resistive torque [35, 65]. DS is recommended to be performed as a controlled movement through the range of motion of the active joint(s) [1, 2, 23]. Although monitored by the researchers, youthful exuberance might have progressed these DS to less controlled ballistic movements, which possibly led to some degree of muscle damage. Future studies should investigate the potential damaging effect of DS, as the design of the present protocol cannot bring conclusive evidence to the question.

However, impairments were not apparent with 300°.s^-1^ knee flexors isokinetic force and power-related measures. In fact, with DS and SS conditions, the 300°.s^-1^ knee flexors isokinetic force and power-related measures were higher compared to control (main effect for condition). The force velocity relationship illustrates that the greatest forces are exerted at lower velocities with decreasing force output at higher velocities [66–68]. Hence, with lower force and power outputs at 300°.s^-1^, possible DS-induced minor hamstrings muscle damage may not have been apparent. It is widely believed that SS induces performance deficits [1–3, 69]. While the Behm et al. [1] meta-analysis calculated a SS-induced 3.7% weighted mean performance reduction, the individual study data revealed 119 significant performance (i.e. isometric and dynamic strength tests and jump height) reductions, 145 non-significant findings, and 6 significant improvements. Thus, a greater number of SS studies in the literature either provided no significant impairments or actually improved performance. In the present study, DS and SS conditions may have positively impacted neural excitation that offset testing-induced (control condition) decrements. Since EMG is a gross measure of neuromuscular activation, and not linearly related to muscle force [70, 71] any possible increases in neural excitation may not have been apparent. The curvilinear relationship of force and EMG presents an EMG activity plateau at higher forces [70, 71]. Since testing involved maximal isometric and isokinetic contractions, the lack of change in maximal EMG activity is not that surprising.

Main effects for time showed improvements for drop jump performance at post-test and post-24 hours. A main effect for time includes the control condition and thus especially with young participants, the improvements might be attributed to a practice or learning effect with testing.

## Conclusions

In conclusion, testing-induced impairments of 60°.s^-1^ and 300°.s^-1^ knee extensors isokinetic force and power-related deficits at post-test were counterbalanced by DS. The greater range of motion associated with hip flexion DS could have led to some muscle damage that decreased the higher knee flexor forces and power associated with slower contractions at 60°.s^-1^ for up to 24 hours. However, testing-induced decrements of the lower maximum forces exerted with higher velocity contractions were offset by DS and SS. Thus, as recommended by recent studies [72, 73], a full warm-up incorporating appropriate durations of SS (<60 seconds per muscle group) [1–3] can be incorporated since the combination of SS, DS and dynamic activities should not negatively impact power activities. However, DS must be closely monitored to ensure that it is performed under controlled conditions, without pain, through the range of motion. It is recommended that youth need extensive familiarization with novel testing procedures (i.e. isometric and isokinetic dynamometry).

## Supporting information

S1 Dataset Final excel data worksheet. (XLSX)
